# Increased Risk of Tuberculosis in Patients With Type 1 Diabetes Mellitus: Results From a Population-Based Cohort Study in Taiwan

**DOI:** 10.1097/MD.0000000000000096

**Published:** 2014-10-03

**Authors:** Te-Chun Shen, Cheng-Li Lin, Chang-Ching Wei, Wei-Chih Liao, Wei-Chun Chen, Chia-Hung Chen, Chih-Yen Tu, Te-Chun Hsia, Chuen-Ming Shih, Wu-Huei Hsu, Chia-Hsiang Li, Fung-Chang Sung

**Affiliations:** Division of Pulmonary and Critical Care Medicine (T-CS, W-CL, W-CC, C-HC, C-YT, T-CH, C-MS, W-HH, C-HL), Department of Internal Medicine, China Medical University Hospital and China Medical University, Taichung; Division of Pulmonary and Critical Care Medicine (T-CS), Department of Internal Medicine, Chu Shang Show Chwan Hospital, Nantou; Institute of Clinical Medical Science (T-CS, F-CS), College of Medicine, China Medical University; Management Office for Health Data (C-LL, F-CS), China Medical University Hospital, Taichung; Division of Nephrology (C-CW), Department of Pediatrics, China Medical University Hospital and China Medical University, Taichung, Taiwan.

## Abstract

The studies on the risk of tuberculosis (TB) in patients with type 1 diabetes mellitus (T1DM) alone are limited. We examined this relationship using a population-based retrospective cohort study. From claims data of the National Health Insurance system of Taiwan, we identified 5195 patients with T1DM newly diagnosed from 2002 to 2011 and 20,780 randomly selected controls without T1DM, frequency matched by age, sex, and year of diagnosis. Both cohorts were followed up until the end of 2011 to evaluate the risk of TB. The overall incidence of TB was 4.07-fold higher in the T1DM cohort than in the control cohort (1.18 vs 0.29 per 1000 person-years, *P* < 0.001). Compared with the controls, the Cox model estimated adjusted hazard ratios (HRs) of TB in patients with T1DM were greater in men than in women (4.62 vs 3.59) and in adults than in children (4.06 vs 3.37), but not significant. The adjusted HR was much greater for those with comorbidities than those without comorbidities (14.6 vs 1.62, *P* < 0.001). Compared with the controls, the patients with T1DM were also more likely to develop TB with multiple emergency room visits (adjusted HR: 116.1, 95% confidence interval [CI] = 43.8–307.4) or hospitalizations (adjusted HR: 86.5, 95% CI = 33.7–222.4). Patients with T1DM are at elevated risks of developing TB with much higher HRs for those with comorbidities, within the first year of diagnosis, and with frequent emergency cares or hospitalizations.

## INTRODUCTION

Tuberculosis (TB) is an infectious disease caused by *Mycobacterium tuberculosis*, and it remains the most prevalent infectious disease and the major leading cause of death worldwide. Every year, approximately 9 million people develop TB and 1.4 million die from the disease, and over 95% of these patients are from developing countries.^1^ As of 2005, the disease was most prevalent in Africa (28% of all TB cases), while half of all new cases were from 6 Asian countries: Bangladesh, China, India, Indonesia, Pakistan, and the Philippines.^2^ With an incidence of 53 cases per 100,000 people in 2012, TB is also a common infectious disease in Taiwan.^3^

There is increasing evidence about an association between TB and diabetes mellitus (DM). Chen et al^4^ performed a meta-analysis study and reported that the combined prevalence rate of DM among patients with TB was 7.2% in China. Given the complexity of mechanisms underlying DM complications and the numerous pathways involved, it is likely that the immune response to *M tuberculosis* infection is affected at multiple levels.^5^ The greatest increases in the prevalence of DM are occurring in developing countries where TB is also endemic.^6^

The effects of DM on TB are complicated. There are differences between TB patients with DM and those without DM with regard to clinical characteristics, radiologic manifestations, and even response to treatment.^7^ Chen et al^8^ reported that the median delay of treatment for TB was significantly higher in patients with DM than those without DM (25 vs 6 days). In another study, Lee et al^9^ revealed that the presence of DM was independently associated with the risk of TB relapse. Patients with DM comorbidity may pose a greater challenge the control of TB.

Type 1 DM (T1DM) is another form of DM resulting from the autoimmune destruction of insulin-producing beta cells in the pancreas. It was previously known as juvenile diabetes; however, T1DM can also be diagnosed in adults.^10^ There are limited studies on the risk of TB in patients with T1DM.^11,12^ Webb et al^12^ reported a high prevalence of TB in children and adolescents with T1DM. Other studies with small study populations have also shown an increased prevalence of TB in adults with T1DM.^13,14^ Most studies on the association between T1DM and TB were case–control studies, and the findings may not be a valid reflection of the true risk of TB in association with T1DM.

Taiwan’s National Health Insurance (NHI) database is a nationwide, large-scale cohort dataset, which provides reliable data and has been used for various studies over the course of many years.^15–17^ In the present study, we attempted to determine whether there is an increased risk of TB in patients with T1DM using the NHI database in Taiwan.

## METHODS

### Data Source

The NHI program, run by the Bureau of the National Health Insurance (BNHI), is a single-payer program launched on March 1, 1995 that covers approximately 99% of the 23.74 million Taiwanese population. BNHI has authorized the National Health Research Institutes to create the National Health Insurance Research Database (NHIRD) for medical research using the administrative and health claims data generated by the NHI program. NHIRD includes complete inpatient care, ambulatory care, dental care, and prescription drugs and provides researchers with scrambled identification numbers associated with the relevant claim information, which includes the patient’s gender, date of birth, registry of medical services, and medication prescriptions. The present study was an analysis of deidentified secondary data; therefore, no informed consent was required. This study was approved by the Research Ethics Committee of the China Medical University, Taichung, Taiwan (CMU-REC-101-012). Diagnostic codes were according to the format of the International Classification of Disease, 9th Revision, Clinical Modification (ICD-9-CM).

### Subject Selection

The study subjects were identified from 2 subdatasets of the NHIRD. First, the T1DM cohort were identified from the Registry of Catastrophic Illnesses Patient Database (RCIPD), a dataset containing health claims data for the treatment of catastrophic illness, which includes 30 categories of diseases that require long-term care. If the insured has major diseases such as cancer or T1DM, he or she can apply for a catastrophic illness certificate. To reduce the financial hardship associated with the catastrophic illness, the NHI program exempts beneficiaries from obligations for NHI-defined catastrophic illnesses. The T1DM cohort included patients aged <40 years, newly diagnosed with T1DM (ICD-9-CM codes 250.x1 and 250.x3) between 2002 and 2011. The date of diagnosis of T1DM was defined as the index date. Second, the control subjects (the non-T1DM cohort) were identified from the Longitudinal Health Insurance Database 2000 (LHID 2000), a database containing the claims data of a million people randomly sampled from 2000 NHIRD enrollment files. There was no significant difference in gender, age, or health care costs between cohorts in LHID 2000 and all insurance enrollees, as reported by the NHI in Taiwan. For each T1DM case, 4 non-T1DM controls frequency matched to the case with regard to gender and the year of T1DM diagnosis was identified. People with a history of TB, type 2 DM (T2DM), or with incomplete information were excluded.

### Definitions of End Point, Comorbidities, and Covariates

The study subjects were followed from the index date to the date of TB diagnosis, withdrawal from the insurance program, censoring because of death, or end date of the database (December 31, 2011). For each subject, the records of comorbidities were obtained before the index date, including chronic liver disease (ICD-9-CM code 571), chronic kidney disease (ICD-9-CM code 585), and previous infection(s). Previous infection(s) was defined as infections including sepsis, bacteremia, infective endocarditis, pneumonia, urinary tract infection, liver abscess, biliary tract infection, soft tissue infection, bone and joint infection, osteomyelitis, central nerve system infection, and postoperative infection.

### Statistical Analysis

We described and compared the distributions of gender, age, and comorbidities (%) between the cohorts with and without T1DM using χ^2^ tests. Person-years were calculated as the interval from the index date to the date of TB diagnosis, loss to follow-up, or end of 2011. The incidence densities (per 1000 person-years) were estimated in both the cohorts. Univariable and multivariable Cox proportional hazards regression analyses were used to estimate the hazard ratio (HR) with 95% confidence interval (CI) for developing TB. The multivariable model including gender, age, and comorbidities was performed to estimate the adjusted HR for the T1DM cohort compared with the control cohort. Further analysis was performed to assess the dose response on the risk of TB according to the number of emergency room (ER) visits and/or hospitalizations for T1DM. The cumulative incidences of TB between the T1DM and control cohorts were assessed using the Kaplan–Meier method, and the differences were assessed using a log-rank test. All statistical analyses were performed using the SAS statistical package (version 9.2 for Windows; SAS Institute, Inc, Cary, NC). Statistical significance was accepted at *P* < 0.05.

## RESULTS

**FIGURE 1:**
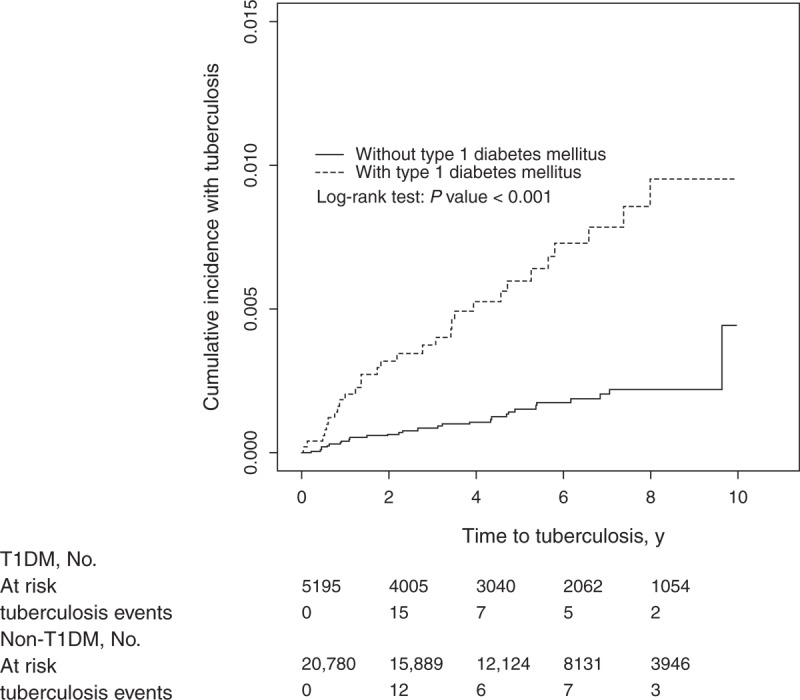
Cumulative incidence of tuberculosis for patients with (dashed line) or without (solid line) type 1 diabetes mellitus.

We identified 5195 patients with T1DM between 2002 and 2011 as the T1DM cohort and 20,780 non-T1DM subjects as the control cohort. Of the 5195 patients with T1DM, 52.3% were female and 56.5% were aged <20 years (Table 1). The mean ages of subjects in the T1DM and control cohorts were 18.9 (±10.2) and 23.8 (±9.81) years, respectively. The subjects in the T1DM cohort tended to have a higher prevalence of chronic liver disease, chronic kidney disease, and previous infection(s) than those in the control cohort. The mean follow-up time was 4.90 (standard deviation [SD] = 2.93) years in the T1DM cohort and 4.83 (SD = 2.92) years in the non-T1DM cohort (data not shown). Figure 1 shows the cumulative TB incidence curve for the 2 cohorts and indicates that the incidence curve of TB was significantly higher in the T1DM cohort than in the control cohort (log-rank test, *P* < 0.001).

**TABLE 1:**
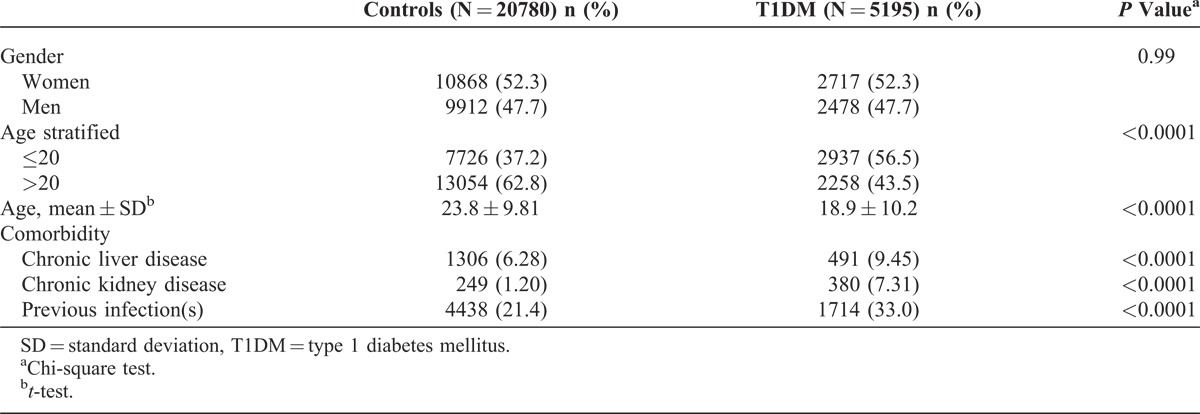
Comparisons in Demographic Characteristics and Comorbidities Between Cohorts With and Without Type 1 Diabetes Mellitus

The follow-up results revealed that the T1DM cohort was 4.07-fold more likely to develop TB than the control cohort (1.18 vs 0.29 per 1000 person-years), with an adjusted HR of 4.23 (95% CI = 2.43–7.36) after controlling for age, gender, and comorbidities (Table 2). The incidence of TB was greater in men than in women in both the cohorts. The gender-specific hazard of TB in the T1DM cohort relative to that in the control cohort was significant for both women (adjusted HR: 3.59, 95% CI = 1.54–8.37) and men (adjusted HR: 4.62, 95% CI = 2.21–9.64). The incidence of TB increased with age in both the cohorts. The age-specific hazard of TB in the T1DM cohort relative to that in the control cohort was significant for all age groups (≤20 years, adjusted HR: 3.37, 95% CI = 1.14–9.94; >20 years, adjusted HR: 4.06, 95% CI = 2.15–7.69). T1DM patients with comorbidity had an adjusted HR of 14.6 (95% CI = 5.50–38.7). Stratified analysis by follow-up duration revealed that the incidence of TB decreased with the follow-up time. The adjusted HR of TB was significantly higher in the first follow-up year (adjusted HR: 5.63, 95% CI = 2.09–15.2) than the later years (adjusted HR: 3.70, 95% CI = 1.90–7.21).

**TABLE 2:**
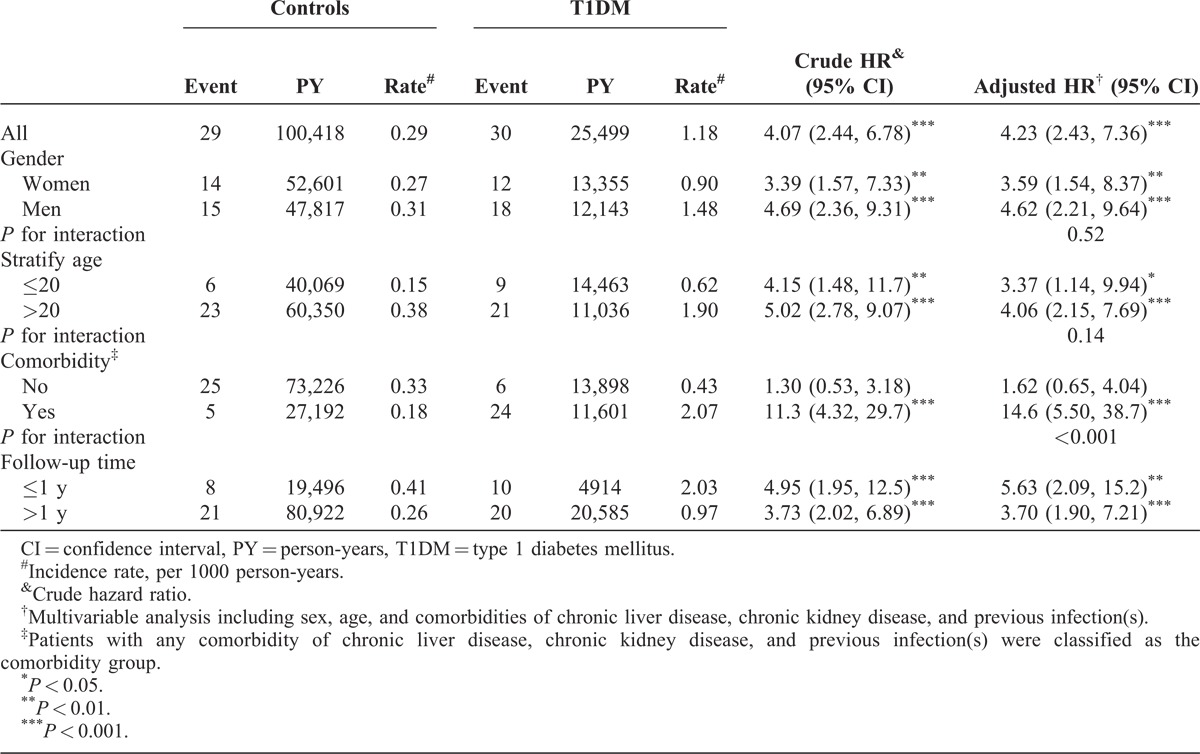
Incidence of Tuberculosis and Cox Method Estimated Hazard Ratio of Tuberculosis for TIDM Cohort Compared With Control Cohort by Demographic Characteristics and Comorbidity

In Table 3, it is shown that the HRs of TB for patients with T1DM associated with the frequency of ER visits and hospitalizations. Compared with the control cohort, T1DM patients with the ER visit for more than twice for their diabetes had an extremely high adjusted HR of 116.1 (95% CI = 43.8–307.4) to develop TB. Meanwhile, the adjusted HR of TB was 86.5 (95% CI = 33.7–222.4) in the patients having T1DM who had been hospitalized for more than twice for their diabetes.

**TABLE 3:**
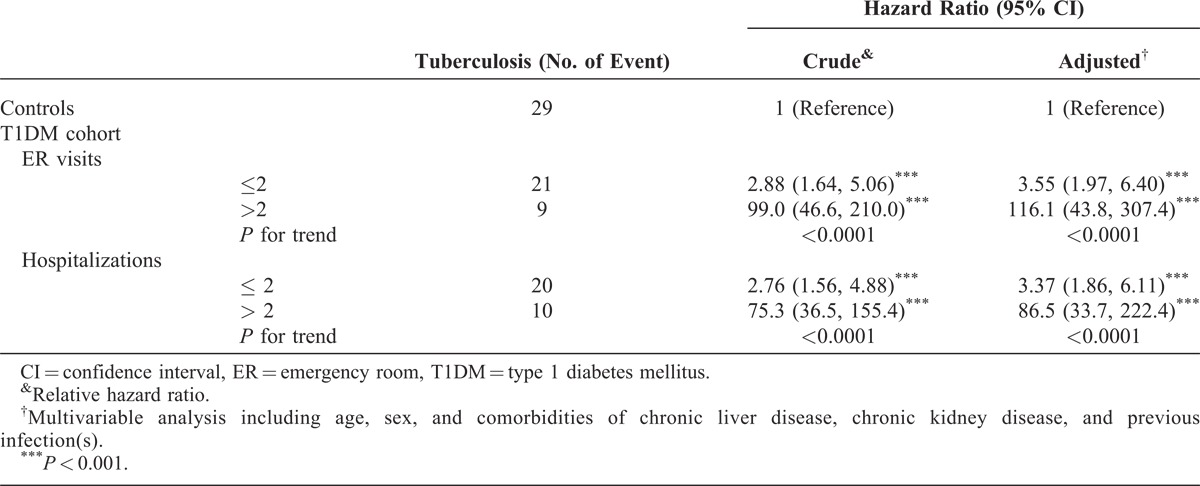
Hazard Ratio of Tuberculosis Associated With the Number of Annual Emergency Room Visits and Hospitalizations for Patients With Type 1 Diabetes Mellitus

## DISCUSSION

The results of our population-based study demonstrated an increased risk of TB in patients with T1DM in comparison with the general population (adjusted HR: 4.23, 95% CI = 2.43–7.36). Further analysis indicated that the risk of TB was higher in those with comorbidities (adjusted HR: 14.6, 95% CI = 5.50–38.7, *P* for interaction <0.001). We also observed that the adjusted HR (5.63, 95% CI = 2.09–15.2) was markedly higher during the initial 12 months following the diagnosis of T1DM. Another important finding is that T1DM patients with a higher number of hospitalizations or ER visits have >85-fold of HR for developing TB (Table 3). To the best of our knowledge, this is the first population-based study revealing such a strong relationship between T1DM and the risk of TB.

Although the precise pathophysiological mechanism underlying the effect of DM as a predisposing risk factor for TB remains unknown, hypotheses that have been suggested included depressed cellular immunity, dysfunction of alveolar macrophages, low levels of interferon gamma, pulmonary microangiopathy, and micronutrient deficiency.^18^ An animal study shows that rats with T1DM are highly susceptible to *M tuberculosis* infection. The alveolar macrophages of rats with T1DM are not completely activated for killing *Tubercle bacilli*, unlike those from normal rats.^19^ In addition, Webb et al^12^ reported that children and adolescents with T1DM appear to have a 6.8-fold higher prevalence of TB, although they did not provide a direct comparison.

Poor glycemic control has been shown to be significantly associated with the prevalence of TB. Several studies have shown that transient or chronic hyperglycemia alters the immune function in patients with DM. The chronic upregulation of glucose leads to the abnormal accumulation of advanced glycation end products (AGE), which can bind and modify immune response molecules.^20^ Excess AGE may promote the accumulation of reactive oxidative species, hence, leading to oxidative stress. Oxidative stress in patients with DM may lead to the reduction of interleukin-12 in response to the infection of *M tuberculosis.*^21^ Patients with DM appear to have alterations in innate immunity with reduced phagocytosis, contributing to the antigen presentation and secretion of antimycobacterial peptides. In regard to adaptive immunity, diabetic patients thus have a hyper-reactive cell-mediated response to *M tuberculosis* antigen.^22,23^

TB patients with DM are quite different from those without DM with regard to clinical characteristics, radiologic manifestations, and even response to treatment. Some studies have reported that TB patients with DM have more symptoms but less extrapulmonary involvement,^24,25^ while other studies have reported a higher incidence of lower lobe involvement among TB patients with DM but no difference in the frequency of pleural effusion or pleural TB.^26,27^ DM is thought to have a negative impact on the outcome of TB treatment, with higher failure rates, higher all-cause mortality rates, and fatality specifically related to TB.^28–30^ Furthermore, DM has been reported to be an independent risk factor for TB relapse.

In a similar study, Kuo et al^6^ used the same NHI database in Taiwan and found that the overall incidence rate of TB was 1.43-fold higher in a T2DM cohort than in a control cohort (2.08 vs 1.45 per 1000 person-years). The mean age of their T2DM cohort is much older than that of our T1DM cohort (55.5 vs 18.9 years). The incidence rates of TB in younger patients are lower in their study than in ours (0.58 in females and 0.79 in males per 1000 person-years vs 0.9 in females and 1.48 in males per 1000 person-years). This finding indicates that younger patients with T1DM have a higher risk of developing TB than those with T2DM.

TB is most prevalent with a high mortality rate among all communicable diseases in Taiwan. In 2011 alone, there were 54.5 newly diagnosed cases and 2.8 deaths per 100,000 persons.^31^ In Taiwan, TB is considered as a class 3 notifiable disease; every newly diagnosed case is required to be reported within a week to the Taiwan Centers for Disease Control. Therefore, the data on the diagnosis of TB are reliable. With regard to credible certification for T1DM, it is categorized as a “catastrophic illness” and patients diagnosed with T1DM are entitled to receive “catastrophic illness certification” issued by the insurance authority. The catastrophic illness-certified patients are eligible for a considerable discount with regard to medical expenses. The certification process requires critical evaluation of medical records and/or serological reports by experts specialized in the disease field. A spot check is also regularly performed. Therefore, NHIRD provides a reliable data source for the occurrence of TB and T1DM.

The strengths of our study is the use of population-based data that are highly representative of the general population. However, certain limitations of this study should be considered. First, NHIRD does not contain detailed information on occupation, smoking habits, alcohol consumption, body mass index, diet preference, physical activity, environmental exposure, drug use, and family history, all of which are potential confounding factors. Second, several relevant clinical variables such as laboratory data, imaging results, culture reports, and pathology findings were not available for the patients included in our study. Although the information on serum blood sugar or glycated hemoglobin level was unavailable, our data suggested that the number of hospitalizations and/or ER visits related to T1DM represented, in part, the blood glucose control status. We found that the number of hospitalizations and/or ER visits had a high correlation with susceptibility to TB. Third, regardless with large sample sizes, the incident TB is the relatively small number at the end of follow-up in this study. Small incidence limits the statistical analyses, particularly regarding covariate adjustments and subgroup analyses, such as for patients with and without several ER visits and hospitalizations. However, the strong association that appears in this study is an important message for populations in other areas with high prevalence of TB.

## CONCLUSION

Patients with T1DM have a significantly higher risk of developing TB than the general population. The risk is much higher in patients with comorbidities and within the first year of diagnosis. A greater attention is required for patients having T1DM with more frequency hospitalizations and ER visits because of a markedly increased risk of developing TB.
